# The risk of oral squamous cell carcinoma in patients with and without somatoform disorders including bruxism: A retrospective evaluation of 309,278 individuals

**DOI:** 10.3389/fonc.2022.1080492

**Published:** 2023-01-09

**Authors:** Marlene Heym, Max Heiland, Robert Preissner, Christopher Huebel, Susanne Nahles, Andrea Maria Schmidt-Westhausen, Saskia Preissner, Moritz Hertel

**Affiliations:** ^1^ Department of Oral and Maxillofacial Surgery, Charité - Universitätsmedizin Berlin, corporate member of Freie Universität Berlin, Humboldt-Universität zu Berlin, and Berlin Institute of Health, Berlin, Germany; ^2^ Institute of Physiology and Science-Information Technology (IT), Charité - Universitätsmedizin Berlin, corporate member of Freie Universität Berlin, Humboldt-Universität zu Berlin, and Berlin Institute of Health, Berlin, Germany; ^3^ Social, Genetic & Developmental Psychiatry Centre, Institute of Psychiatry, Psychology & Neuroscience, King’s College, London, United Kingdom; ^4^ National Centre for Register-based Research, Aarhus Business and Social Sciences (BSS), Aarhus University, Aarhus, Denmark; ^5^ Department of Periodontology, Oral Medicine and Oral Surgery, Charité - Universitätsmedizin Berlin, corporate member of Freie Universität Berlin, Humboldt-Universität zu Berlin, and Berlin Institute of Health, Berlin, Germany

**Keywords:** somatoform disorder, bruxism, oral squamous cell carcinoma, oral cancer, OSCC

## Abstract

**Background:**

The question arises if there is an association of psycho-emotional stress and chronic soft tissue injuries caused by bruxism somatoform disorders with oral squamous cell carcinoma (OSCC).

**Methods:**

Patients with and without “somatoform disorders including psychogenic disturbances” (International Classification of Diseases [ICD]-10 code F45.8), and/or “unspecific behavioral syndromes” (F59), and/or “sleep related bruxism” (G47.63), and/or “other sleep disorders” (G47.8) were retrieved from the TriNetX network to gain cohort I. Cohort II was formed by patients without the aforementioned diagnoses, and by matching for age, gender, tobacco use, and alcohol abuse. After defining the primary outcome as “OSCC” (ICD-10 codes C00−C14), a Kaplan-Meier analysis was performed, and risk ratio (RR) and odds ratio (OR) were calculated.

**Results:**

After matching, each cohort accounted for 154,639 patients (59.7% females; 40.3% males; mean current age (± standard deviation) = 43.4 ± 24.5 years). Among cohorts I and II, 907 and 763 patients, respectively, were diagnosed with OSCC within 5 years (risk of OSCC = 0.6% and 0.5%), whereby the risk difference was significant (*p* < 0.001; Log-Rank test). RR and OR were 1.19 (95% confidence interval (CI), lower = 1.08 and upper = 1.31) and 1.19 (95% CI, 1.08−1.31).

**Conclusions:**

Psycho-emotional stress and/or chronic mucosal injuries may play a role in carcinogenesis. However, the results need to be interpreted cautiously due to limitations of the applied approach. It may thus far only be concluded that further research is necessary to investigate hypotheses regarding psychogenic carcinogenesis and tumor formation due to chronic tissue trauma.

## Background

Oral squamous cell carcinoma (OSCC) is the most frequent malignant tumor of the head and neck region (1, 2). The worldwide incidence is over 300,000 per year (1). The risk of both cancer within the oral cavity and the larynx is augmented by tobacco use and alcohol consumption. Oropharyngeal carcinoma was furthermore shown to be related with human papillomavirus (HPV), specifically the subtypes 16 and 32. The underlying mechanisms of cancer development are relatively well understood, based on knowledge about key fundamental cellular alterations and evidence of histopathological progression from epithelial atypia through increasing stages of dysplasia (3, 4). Accordingly, several entities and conditions providing an augmented risk of OSCC have been identified and classified as oral potentially malignant disorders (OPMD) by the World Health Organization (WHO) (5).

Somatoform disorders (including psychogenic disturbances [International Classification of Diseases 10 [ICD-10] code F45.8]) are mental or physical disturbances which lack an organic basis. They can present as either dissociation (with impaired memory, consciousness and self-identity) or disturbances with somatization (6). The etiopathogenesis of somatoform disorders may be characterized as extremely complex as it includes genetic, neurological, neurophysiological, psychophysiological, endocrinological, perceptive, as well as cognitive aspects, and social learning, knowledge of illness, public conceptualization of illness, negative life events, chronic psychosocial stress, reduced coping skills, lacking supporting systems, systems of social reinforcement, coexistence/comorbidity of psychiatric disorders, traumapsychology, and psychodynamics play a potential role (7). In that context behavioral syndromes associated with psychological disturbances and physical factors (F59) are correlated behaviors expressed within a given behavioral context, e.g., autoaggressiveness (8). Sleep related bruxism (G47.63) as a specific somatoform disorder is defined as grinding of teeth due to uncontrolled activity of certain masticatory muscles during sleep (9). It is considered as a sleep-related movement disorder with a multifactorial etiology including psychological causes (10). Together with other sleep disorders (G47.8) bruxism was shown to correlate with an impaired (oral) health-related quality of life (11, 12). Furthermore, epidemiological as well as experimental studies provide evidence for psycho-emotional stress playing a relevant role in carcinogenesis (13–18). Chronic emotional distress was shown to increase the release of stress hormones and endogenous mutagens, such as reactive oxygen and nitrogen species (ROS and RNS), which in turn can damage deoxyribonucleic acid (DNA) and alter immune function. Hence, psycho-emotional stress seems to be capable of activating key mechanisms of carcinogenesis, which can be conflated as psychogenic carcinogenesis hypothesis (19). Emotional distress activating the hypothalamus-pituitary-adrenalin axis, as well as the sympathetic nervous system, was furthermore suspected to aid in the progression of oral cancer (20).

Despite being related with psycho-emotional stress, bruxism was discussed as contributing to oral cancer through chronic soft tissue injuries triggering the carcinogenesis process (21, 22). Evidence was reported that chronic trauma of the oral mucosa is associated with an increased frequency of OPMDs and OSCC (23). Despite having been found to potentially mimic malignant lesions, oral self-mutilation has not yet been proven to contribute to OSCC (24). Even though bruxism is known to cause hyperkeratosis of the mucous membranes it must be emphasized that this is not a criterion for dysplasia, at least not in the case of the buccal mucosa where respective lesions are most found. Accordingly, bruxism is not classified as OPMD according to the WHO (5).

The aim of the present study was to investigate the risk of developing OSCC in subjects suffering from somatoform disorders, including psychogenic disturbances (e.g., bruxism and dysphagia), and/or unspecific behavioral syndromes associated with psychological disturbances and physical factors, and/or sleep-related bruxism, and/or other sleep disorders in comparison with individuals without the respective diagnoses. Even though somatoform disorders, including psychogenic disturbances, are not classified as predisposing factors for oral cancer (5), it was hypothesized that the risk of OSCC was higher in patients with the aforementioned diseases than in subjects without somatoform disorders.

The TriNetX Global Health Research Network (TriNetX, Cambridge, Massachusetts, USA) was selected to gain data on the subject, as it provides access to a significant number of medical records from more than 67 health care organizations (HCOs) in 11 countries. TriNetX is a so-called real-world database. Its intent is to bring together HCOs, contract research institutes, and pharmaceutical companies to collect and exchange longitudinal clinical data and to provide state-of-the-art statistical analytics. By January 2022, TriNetX had collated electronical medical records of more than 250 million individuals. The network has previously been used to research medical topics of global importance, including the coronavirus disease 2019 (COVID-19) pandemic (25, 26).

## Patients and methods

### Data acquisition, inclusion and exclusion criteria, allocation, and matching

The TriNetX database was searched for individuals who visited the HCO for “inpatient encounter”, and who met the index event in terms of having been diagnosed with either “somatoform disorders including psychogenic disturbances” (e.g., bruxism and dysphagia; ICD-10 code F45.8), and/or “unspecific behavioral syndromes associated with psychological disturbances and physical factors” (ICD-10 code F59), and/or “sleep related bruxism” (ICD-10 code G47.63), and/or “other sleep disorders” (G47.8) based on the ICD-10. Only medical records which covered at least five years of follow-up were included. Furthermore, the eligibility period was limited to the past 20 years from the access date. Thus, older records were excluded from the analysis. The obtained patients were assigned to cohort I (= subjects diagnosed with ICD-10 codes F45.8, and/or F59, and/or G47.63, and/or G47.8 within the past 20 years). Cohort II was formed by retrieving individuals from the database who had not been diagnosed with ICD-10 codes F45.8, and/or F59, and/or G47.63, and/or G47.8 within the past 20 years, and whose medical records covered at least five years of follow-up after visiting the HCO for inpatient encounter. Finally, one-to-one matching for age and gender distribution, as well as tobacco use and alcohol abuse, was performed to mitigate confounder bias *via* the propensity score, and to replicate randomized conditions as closely as possible.

### Data analysis

The primary outcome was defined as “OSCC” (ICD-10 codes C00−C14). Additionally, the time window was set to 5 years after the index event (diagnosis of ICD-10 codes F45.8, and/or F59, and/or G47.63, and/or G47.8 in the case of cohort I and visit of the HCO for inpatient encounter in the case of cohort II). Outcome events were recorded on a daily interval. Subsequently, a Kaplan-Meier survival analysis was performed, and risk ratio (RR) and odds ratio (OR) were determined. Statistical analysis was performed using the Log-Rank test, whereby *p* ≤ 0.05 was defined as significant.

## Results

### Assessment, allocation, and matching

**Figure 1:**
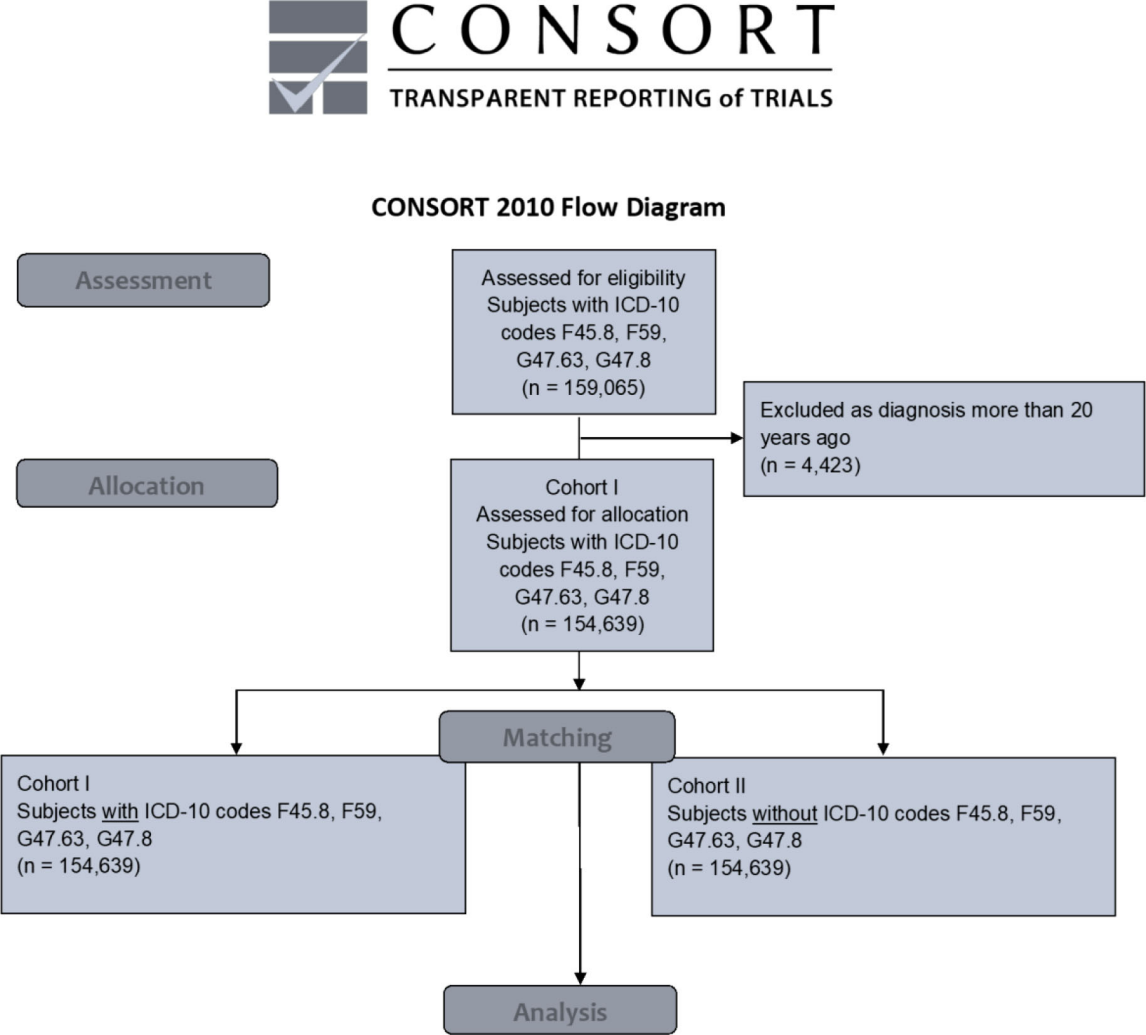
Modified CONSORT flow chart. ICD-10 code F45.8 = “somatoform disorders including psychogenic disturbances” (e.g., bruxism and dysphagia), F59 = “unspecific behavioral syndromes associated with psychological disturbances and physical factors”, G47.63 = “sleep related bruxism”, G47.8 = “other sleep disorders” ICD-10 = International Classification of Diseases 10 OSCC = oral squamous cell carcinoma.

The access date was July 21, 2022. For having met the index event more than 20 years before the access date, 4,423 patients diagnosed with ICD-10 codes F45.8, and/or F59, and/or G47.63, and/or G47.8 were excluded from the analysis. After matching, each cohort accounted for 154,639 individuals (cohort I = females: 92,357 [59.7%]; males: 62,382 [40.3%]; mean current age = 43.4 ± 24.5 years; cohort II = females: 92,358 [59.7%]; males: 62,381 [40.3%]; mean current age = 43.3 ± 24.5 years), as shown in the modified CONSORT diagram (**Figure 1**) and in **Table 1**. Age and gender distribution, as well as the frequencies of tobacco smoking and alcohol abuse, did not differ significantly between the cohorts (*p* > 0.05). Propensity score was 0.98. Data were obtained from 55 HCOs.

**Table 1:** Characteristics of the cohorts I (individuals with ICD-10 codes F45.8, and/or F59, and/or G47.63, and/or G47.8) and II (subjects without the respective diagnoses) before and after matching for age and gender, as well as tobacco use and alcohol abuse.

	Before matching				After matching			
Patients (n)	Cohort I	Cohort II	*p*-value	Standardized mean difference	Cohort I	Cohort II	*p*-value	Standardized mean difference
Total	154,642	16,956,753			154,639	154,639		
Males	62,282 (40.3%)	7,479,263 (45.1%)			62,282 (40.3%)	62,281 (40.3%)		
Females	92,360 (59.7%)	9,477,490 (55.9%)	< 0.001	0.078	92,357 (59.7%)	95.358 (59.7%)	< 0.001	0.99
Mean age	43.4	45.7	< 0.001	0.098	43.3	43.3	< 0.001	1.0
Standard deviation	24.5	24.4			24.5	24.5		
Tobacco use	26,619 (17.2%)	530,511 (3.1%)	< 0.001	0.479	26,616 (17.2%)	26.616 (17.2%)	< 0.001	0.99
Alcohol abuse	5,435 (3.5%)	96,736 (0.6%)	< 0.001	0.21	5,432 (3.5%)	5.432 (3.5%)	< 0.001	0.93

ICD-10 code F45.8 = “somatoform disorders including psychogenic disturbances” (e.g., bruxism and dysphagia), F59 = “unspecific behavioral syndromes associated with psychological disturbances and physical factors”, G47.63 = “sleep related bruxism”, G47.8 = “other sleep disorders” ICD-10 = International Classification of Diseases.

### Diagnosis of OSCC

**Figure 2:**
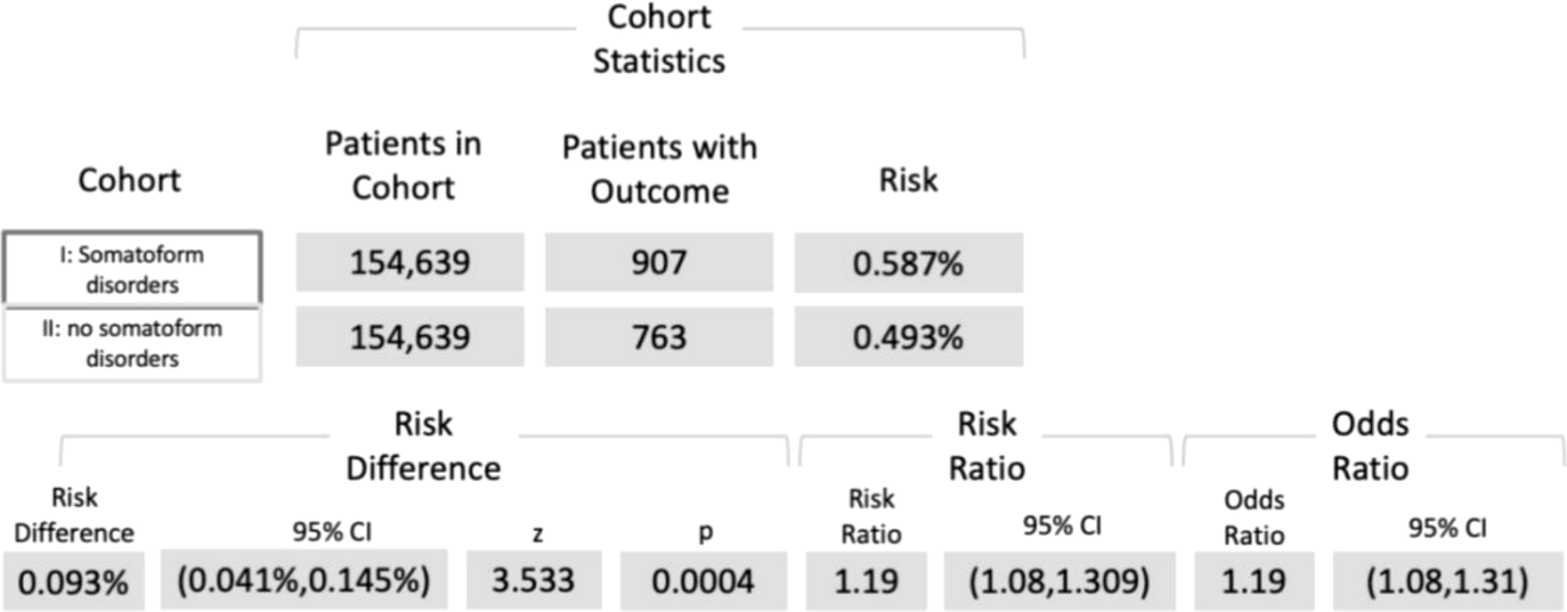
Five-year risk of developing OSCC among patients diagnosed with ICD-10 codes F45.8, and/or F59, and/or G47.63, and/or G47.8 (cohort I) compared with individuals without the respective disturbances (cohort II). ICD-10 code F45.8 = “somatoform disorders including psychogenic disturbances” (e.g., bruxism and dysphagia), F59 = “unspecific behavioral syndromes associated with psychological disturbances and physical factors”, G47.63 = “sleep related bruxism”, G47.8 = “other sleep disorders” ICD-10 = International Classification of Diseases 10.

Among the cohorts I and II, 907 and 736 subjects, respectively, were diagnosed with OSCC (ICD-10 codes C00−C14). The associated risk of developing oral cancer was 0.6% and 0.5%, respectively, whereby the risk difference was highly significant (*p* < 0.001; Log-Rank test). RR and OR were 1.19 (95% CI, 1.08−1.31) and 1.19 (95% CI, 1.08−1.31), as displayed in **Figure 2**.

## Discussion

The intent of the study was to assess if the risk of developing OSCC differed between subjects suffering from somatoform disorders, including psychogenic disturbances (e.g., bruxism and dysphagia), and/or unspecific behavioral syndromes associated with psychological disturbances and physical factors, and/or sleep related bruxism, and/or other sleep disorders and individuals without the aforementioned diagnoses. It was assumed that the risk of OSCC was higher in patients with somatoform disorders than in subjects without the respective disturbances. The hypothesis was confirmed, so far. Within a five-year time window individuals with somatoform disorders revealed an augmented risk of developing OSCC compared to subjects without psychogenic disturbances. Even though the underlying risk difference was found to be only 0.1%, statistical testing revealed a high significance (*p* < 0.001). OR and RR were both 1.19, whereby the obtained confidence intervals substantiate the consistency of the obtained results.

Somatoform disorders, including psychogenic disturbances, are related with psycho-emotional distress, which in turn was suspected to potentially contribute to carcinogenesis (13–17, 27). Furthermore, bruxism may promote cancer development through chronic mechanical injuries of certain parts of the oral mucosa (21, 22). Both mechanisms may have led to the obtained results by contributing to OSCC in cohort I. Despite these considerations that led to the aforementioned assumption, it has to be emphasized that the WHO does not classify “somatoform disorders including psychogenic disturbances” (e.g., bruxism and dysphagia; ICD-10 code F45.8), “unspecific behavioral syndromes associated with psychological disturbances and physical factors” (F59), “sleep related bruxism” (G47.63), nor “other sleep disorders” (G47.8) as predisposing factors for oral cancer (5). Furthermore, the concepts of psychogenic carcinogenesis, as well as cancer development through chronic mechanical trauma, are controversial discussions. Both ideas might be best characterized as hypotheses, even though there is certain supportive evidence. However, the impact of somatoform disorders including bruxism on the risk of oral cancer has not yet been addressed in the recent literature. Basically, the aforementioned disorders are not classified as risk factors for OSCC due to limited evidence. Thus far, the retrieved results support the underlying assumptions, and may encourage future research on the topic. Nevertheless, the obtained results need to be interpreted cautiously.

Another potential focus of further studies could be frequent screening of patients suffering from OSCC for the necessity of psycho-oncological treatment, as only in a minority of subjects is cancer therapy accompanied by psychological treatment (28). Regarding the presented results, it might be further hypothesized that supportive psychotherapy improves patient outcomes.

### Study limitations

Alongside the applied method comes specific limitations that need to be addressed regarding the obtained results. As no detailed data on tobacco use (pack-years), alcohol abuse (consumed alcohol units), and HPV status were available, a certain risk of confounder bias should be considered. Distribution differences tend to be leveled-out to a certain extent in large cohorts, and by matching. However, the proportion of heavy smokers and heavy alcohol consumers could have been lager among the patients with somatoform disorders than among the individuals without the respective disorders. This might have led to a higher risk of OSCC within cohort I. Ohayon et al. (2001) found that sleep bruxism was more frequently found among smokers (OR = 1.3) and heavy alcohol drinkers (OR = 1.8), but also in subjects with a highly stressful life (OR = 1.3) and those with anxiety (OR = 1.3). These findings suggest that different variables might have an influence on the researched question, but these factors may also act as confounders, as expressed above. Future studies may apply a prospective approach to involve data especially on smoking behavior, alcohol consumption, HPV status, but also on stress levels. Furthermore, the exact tumor localization could not be retrieved from the database. A relation with mechanical alteration of the mucous membranes therefore remains uncertain, as bruxism is capable of causing trauma to the buccal mucosa, the tongue, and the lips. OSCC of other localizations can clearly not be directly attributed to bruxism.

### Clinical relevance

It can thus far be concluded that the retrieved results indicate an augmented risk of OSCC among patients with certain somatoform disorders, including bruxism. The findings need to be interpreted cautiously regarding the limitations discussed above. At a minimum, the harvested data support the hypotheses of psychogenic carcinogenesis as well as tumor development based on chronic mechanical traumatization. This might encourage further research. If the presented results could be confirmed in the future, somatically-focused cancer therapy may be supplemented with approaches focusing on psychogenically-induced carcinogenesis. Furthermore, preventive strategies may imply diagnosis and therapy of somatoform disorders.

## Data availability statement

The datasets used and analyzed can be retrieved from the TriNetX network (https://trinetx.com). If no access is available, the datasets can be retrieved from the corresponding author based on reasonable request.

## Ethics statement

Ethical review and approval was not required for the study on human participants in accordance with the local legislation and institutional requirements. Written informed consent from the [patients/ participants OR patients/participants legal guardian/next of kin] was not required to participate in this study in accordance with the national legislation and the institutional requirements.

## Author contributions

Conceptualization: SP, MarH. Methodology: SP, RP, MorH. Software: SP, RP. Validation: MarH, MaxH, SN, CH, AS-W, MorH. Data curation: SP, RP, MarH, MorH. Writing original draft: MarH, SP, MorH. Writing, Review, Editing: MaxH, RP, CH, SN, AS-W. Visualization: RP, SP, MorH. Project Administration: SP, MaxH. Supervision and funding acquisition: RP. All authors read and approved the final manuscript. All authors contributed to the article and approved the submitted version.
